# Cost-conscious generation of multiplexed short-read DNA libraries for whole-genome sequencing

**DOI:** 10.1371/journal.pone.0280004

**Published:** 2023-01-27

**Authors:** Ashley Jones, David Stanley, Scott Ferguson, Benjamin Schwessinger, Justin Borevitz, Norman Warthmann

**Affiliations:** 1 Research School of Biology, Australian National University, Canberra, ACT, Australia; 2 Diversity Arrays Technology, Bruce, ACT, Australia; 3 Plant Breeding and Genetics Laboratory (PBGL), Joint FAO/IAEA Center of Nuclear Techniques in Food and Agriculture, International Atomic Energy Agency (IAEA), IAEA Laboratories, Seibersdorf, Austria; Julius Kuhn-Institut, GERMANY

## Abstract

Massively parallel, second-generation short-read DNA sequencing has become an integral tool in biology for genomic studies. Offering highly accurate base-pair resolution at the most competitive price, the technology has become widespread. However, high-throughput generation of multiplexed DNA libraries can be costly and cumbersome. Here, we present a cost-conscious protocol for generating multiplexed short-read DNA libraries using a bead-linked transposome from Illumina. We prepare libraries in high-throughput with small reaction volumes that use 1/50^th^ the amount of transposome compared to Illumina DNA Prep tagmentation protocols. By reducing transposome usage and optimising the protocol to circumvent magnetic bead-based clean-ups between steps, we reduce costs, labour time and DNA input requirements. Developing our own dual index primers further reduced costs and enables up to nine 96-well microplate combinations. This facilitates efficient usage of large-scale sequencing platforms, such as the Illumina NovaSeq 6000, which offers up to three terabases of sequencing per S4 flow cell. The protocol presented substantially reduces the cost per library by approximately 1/20^th^ compared to conventional Illumina methods.

## Introduction

Massively parallel short-read DNA sequencing, known as second- or next-generation sequencing, enabled an unprecedented increase in sequencing scale and affordability compared to first-generation, electrophoresis based technologies [1]. Second-generation sequencing technologies, such as Illumina platforms, adopted approaches such as sequencing by synthesis, incorporating fluorescent reversible terminator deoxyribonucleotides during base extension, which occurs in parallel for millions to billions of barcoded fragments at a time [2, 3]. This approach is highly accurate (≥ 99.9%) and enabled exponential increases in the scale of sequencing, for instance the Illumina NovaSeq 6000 offers up to three terabases of 150 bp paired-end sequencing per S4 flow cell [4]. However, to take advantage of this large-scale sequencing platform, high-throughput DNA libraries are needed, which can be costly and cumbersome with large sample numbers, potentially being a financial barrier. Multiple library preparation methods exist, however, tagmentation using transposomes (Tn5 transposase homodimers) has seen widespread adoption [5]. This method utilises the transposome to insert adapters throughout DNA fragments, which are later amplified by PCR using dual index primers [5]. Further developments from Illumina have improved this method, with new transposases that increase genome coverage uniformity by reducing biases [6]. Researchers have since reduced tagmentation reaction volumes and streamlined the procedure to reduce costs, enabling high-throughput genome-wide studies [7–9]. However, more recently, transposomes conjugated directly to magnetic beads have been introduced, offering further improvements in library preparation, such as enabling more variability in DNA input and reducing variability of library fragment sizes [10]. This method is now becoming dominant in the market and previous protocol workflows are either obsolete or require modifications.

We developed and present here a cost-conscious protocol for high-throughput generation of multiplexed short-read DNA libraries for whole-genome sequencing. We focused on Illumina sequencing platforms, which continue to dominate second generation sequencing [1, 2, 4], and we utilise the Bead-Linked Transposome (BLT) now offered by Illumina in DNA Prep kits [10]. To substantially reduce reagent cost in our protocol, we perform the tagmentation reaction in small volumes with 1/50^th^ the amount of the transposome compared to Illumina DNA Prep tagmentation protocols. To streamline the high-throughput workflow (while also further reducing cost and time), we proceed from tagmentation directly to PCR, circumventing magnetic bead-based clean-ups. To achieve this, we make a custom tagmentation buffer that excludes unnecessary hazardous solvents such as dimethylformamide and utilise a crowding agent, polyethylene glycol [5]. In our protocol, the only Illumina component required is the BLT, available separately in Illumina DNA Prep kits. Other reagents utilised are laboratory made buffers, third-party PCR components such as the high-fidelity Q5 DNA polymerase from New England Biolabs (NEB) and we present our own dual index primers to multiplex up to nine 96-well microplates. Using this protocol, we have generated thousands of whole-genome libraries for *Eucalyptus* trees, some of which have been used to explore landscape genomic variation [9]. We have also used this protocol in *Puccinia* fungi genomics, for base correction of long-read *de novo* genome assemblies [11, 12]. The presented protocol is an update to our previous version available on Protocols.io [8] (where both are available), which utilised the non-bead linked transposomes that are now becoming obsolete.

## Methods

The protocol described in this article is published on Protocols.io; https://doi.org/10.17504/protocols.io.14egnx27zl5d/v2.

Supplemental files are also included, which contain custom dual index primers, program files for automated workstations (with descriptions) (PerkinElmer), excel files for converting fluorescent microplate readings to concentrations and a comparison of protocol prices.

### Expected results

**Fig 1:**
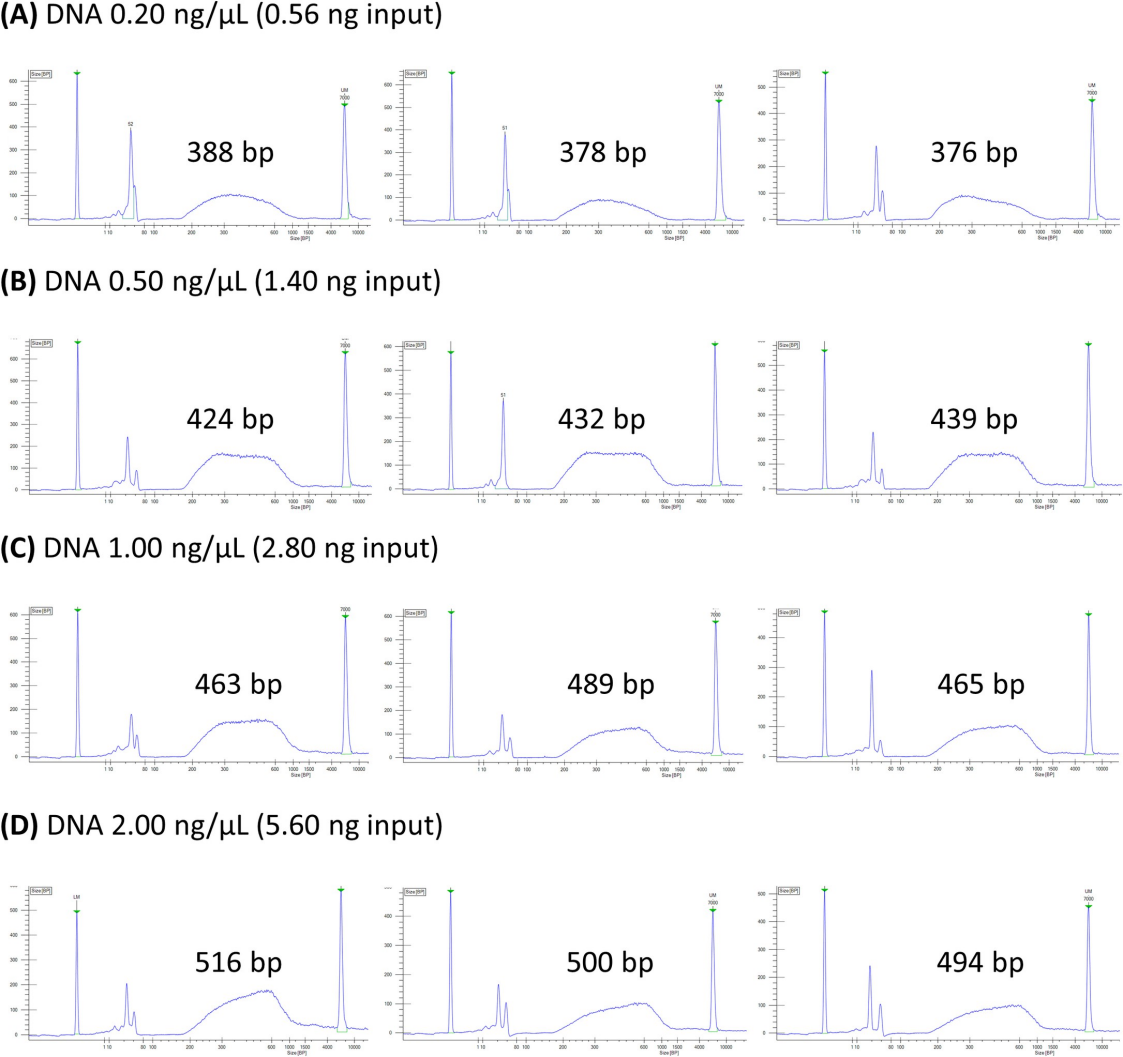
Distribution of fragment sizes for libraries generated with the presented cost-conscious protocol. Libraries were made for independent *Eucalyptus viminalis* samples with varying DNA inputs into the protocol. (A) Four samples with a starting DNA concentration of 0.20 ng/μL each (total 0.56 ng input each). (B) 0.5 ng/μL (1.40 ng input). (C) 1.00 ng/μL (2.80 ng input). (D) 2.00 ng/μL (5.60 ng input). Samples were tested on a LabChip GX Touch HT Nucleic Acid Analyzer (PerkinElmer), using high sensitivity reagents and LabChip according to the manufacturer’s instructions. Electropherogram plots size (bp) against fluorescence intensity and the average library size has been added (excluding the peaks of primers). LM and UM denote the LabChip lower and upper markers respectively (first and last peaks). Note the dual index primers and tagmentation adapters add 136 bp to the amplified library length, therefore library sizes approximately 436 bp are ideal for 150 bp paired-end sequencing.

Using the cost-conscious protocol presented, we have been routinely generating multiplexed libraries in high-throughput for various plant, fungi and metagenome samples. The protocol uses 1/50^th^ the quantity of the transposome (Illumina BLT) for tagmentation compared to Illumina DNA Prep protocols, which reduces library cost substantially (Table 1). This saving on reagent cost combined with protocol optimisations and our own dual index primers reduces the cost per library by approximately 1/20^th^ compared to the Illumina DNA Prep protocol. This helps facilitate research into non-model organisms where funding can be limited. Libraries suitable for sequencing were created under varying DNA inputs, including DNA concentrations low as 0.20 ng/μL (0.56 ng input into a reaction with 0.20 μL transposome) (Fig 1). A starting DNA concentration of 1.00 ng/μL (total 2.80 ng DNA input) appeared the most suitable DNA:transposome optimisation for 150 bp paired-end (300 cycles) sequencing with Illumina. For sequencing of the libraries, we have been utilising the current Illumina platforms, including Illumina MiSeq, NextSeq 500 and NovaSeq 6000. The NovaSeq 6000 is the current leading platform, for which we achieve the expected sequencing outputs across multiple flow cell types for 150 bp paired-end sequencing. For instance, 0.40–0.50 Tbp for S1, 1.00–1.25 Tbp for S2 and 2.40–3.00 Tbp for S4 flow cells. To maintain the required coverage between samples when multiplexing one or more 96-well microplates, we split flow cells into lanes.

**Table 1:** Price comparison between the Illumina DNA Prep protocol and our presented cost-conscious protocol, per sample reaction when performed in high-throughput (96-well microplates). The key difference, the volume of bead-linked transposome (BLT) is also presented. Prices calculated from Australian retail prices (as of July 2022) and presented in the Australian dollar (AUD) and the United States Dollar equivalent (USD to AUD rate of 1.47 as of July 2022). Standard laboratory consumables (such as 96-well microplates, filter tips and microfuge tubes) were excluded, being considered equal in expenditure between the two protocols. Further pricing details are provided in the supplementary material.

Components	Illumina DNA Prep protocol AUD $	Equivalent USD $	Cost-conscious protocol AUD $	Equivalent USD $
Initial DNA quantification	NA	-	$0.20	$0.14
Tagmentation	$66.17	$45.00	$1.32	$0.90
Transposome (BLT) used per reaction	10 μL	-	0.20 μL	-
Custom tagmentation buffer	NA	-	< $0.01	< $0.01
PCR amplification	Included	-	$1.28	$0.87
Dual index primers	$10.71	$7.28	$0.24	$0.16
Post library quantification	Not specified	-	$0.20	$0.14
Clean-up and size selection	Limited	-	$0.51	$0.35
**Total $**	**AUD $76.88**	**USD $52.28**	**AUD $3.75**	**USD $2.55**

**Fig 2:**
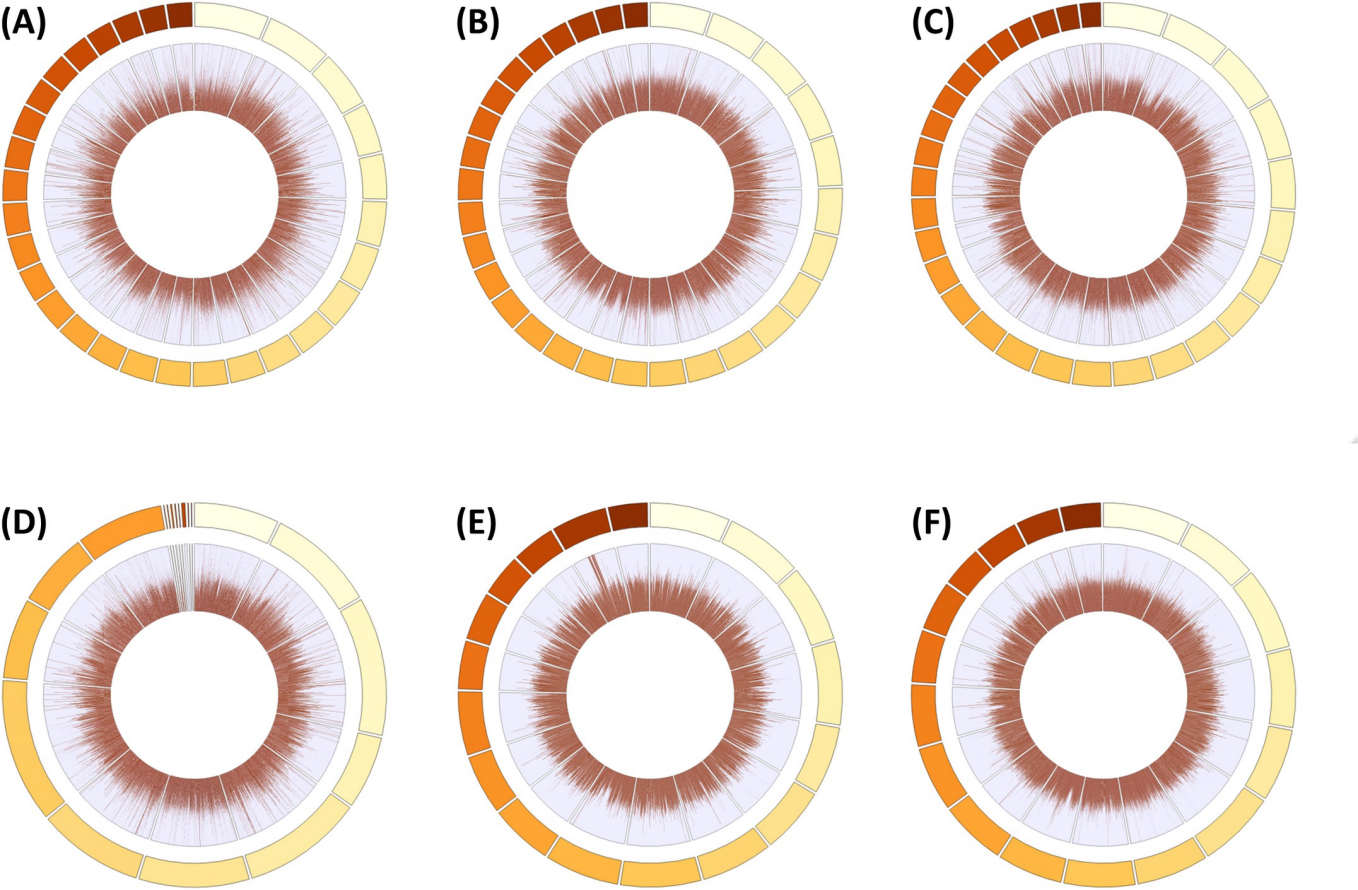
Coverage density of four cost-conscious short-read sequencing libraries generated with the presented protocol, across a corresponding *de novo* genome assembly independently generated with long-read sequencing. (A) *Acacia acuminata*. (B) *Angohpora floribunda*. (C) *Corymbia maculata*. (D) *Eucalyptus melliodora*. (E) *Puccinia striiformis* f. sp. *tritici*. (F) *Puccinia triticina*. Inner circumference represents coverage density, outer circumference represents the genome. Plots were generated by aligning the short-reads with BWA (v0.7.17) [18] to the corresponding long-read genome and calculating per-base read coverage with SAMtools (v1.12, depth tool, -a) [19]. Average coverage per 10 kb bins was calculated with BWA and plotted with the R package BioCircos [20]. A maximum of 30 largest (most contiguous) sequences for each assembly were plotted for visualisation.

Investigating the sequencing data generated from these libraries for selected samples (Table 2), we observe highly accurate raw reads, which meet the Illumina quality standards of ≥ Q30 (99.9%). PCR duplicates were low, < 1% for plants, however, more duplicates were seen in dikaryotic fungi with small genomes, particularly when sequencing coverage was excessive. We confirmed that the expected coverage (an estimate based on sequencing output divided by genome size) is in strong agreement with the observed coverage of mapped reads across a *de novo* genome assembly independently generated with long-read sequencing (Table 2). The mapping quality of these reads were high, > Q40 (99.99%) on average. We saw a high standard deviation in the observed coverage, which reflected the difficulty in mapping short-reads to repetitive DNA loci and fragmented genome assemblies. For example, *P*. *striiformis* f. sp. *tritici* was the most challenging, which is unsurprising given 40% of the genome is estimated to be comprised of repeats [11]. This reduced the coverage at some loci and conversely others were increased. To further investigate, we plotted the coverage density of selected libraries across the genome assembly (Fig 2). We saw distribution of coverage across the whole-genome and the minority of loci with variable coverage was confirmed to be repetitive DNA loci and potential errors in the genome assembly. This provided strong confidence in our cost-conscious protocol in generating whole-genome sequencing data for genomic research.

**Table 2:** Summary statistics of six libraries generated with the presented cost-conscious protocol and sequenced on the Illumina NovaSeq 6000 platform. Libraries consist of four plants (*Acacia acuminata*, *Angohpora floribunda*, *Corymbia maculata*, *Eucalyptus melliodora*) and two fungi (*Puccinia striiformis* f. sp. *tritici*, *Puccinia triticina*). Quality scores are presented based on the Phred scale. PCR duplicates were calculated independent of a reference genome, with HTStream SuperDeduper [17]. Expected coverage was estimated by sequencing output divided by genome size. Observed coverage was calculated by mapping the reads against an independently generated long-read *de novo* assembly. SD denotes standard deviation.

Sample	Genome size (approx.)	Raw read quality (average ± SD)	PCR duplicates (raw reads)	Expected coverage (estimate)	Observed coverage (average ± SD)	Mapping quality (average ± SD)
*A*. *acuminata*	881 Mbp	Q35.94 ± 2.08	0.08%	17.53	16.93 ± 97.20	Q42.21 ± 10.20
*A*. *floribunda*	389 Mbp	Q35.95 ± 2.07	0.13%	48.14	46.32 ± 284.70	Q50.00 ± 11.84
*C*. *maculata*	404 Mbp	Q35.94 ± 2.07	0.08%	38.12	36.83 ± 188.96	Q51.30 ± 10.76
*E*. *melliodora*	639 Mbp	Q35.95 ± 2.07	0.08%	30.22	28.96 ± 99.57	Q49.95 ± 9.01
*P*. *striiformis*	80 Mbp	Q34.63 ± 3.14	25.87%	114.20	87.23 ± 804.25	Q36.24 ± 18.41
*P*. *triticina*	124 Mbp	Q34.67 ± 3.18	19.46%	72.32	59.79 ± 311.20	Q47.28 ± 5.99

Using the protocol presented and sequencing data generated, we have been able to investigate landscape genomic variation in Australian trees *Eucalyptus albens* and *Eucalyptus sideroxylon* [9]. The presented protocol also complements our high-molecular weight DNA protocol [13], as *de novo* genome assembly with long-reads often require base-correction (polishing) with Illumina short-reads [14]. For instance, we have used short-read libraries generated with this protocol to improve base quality of Oxford Nanopore Technologies long-read assemblies in three *Eucalyptus* species [15], wild rice *Oryza australiensis* [16], wheat stripe rust fungus *Puccinia striiformis* f. sp. *tritici* [11] and wheat leaf rust fungus *Puccinia triticina* [12]. Many of the DNA sequencing datasets generated with this protocol are being made available on the National Center for Biotechnology Information (NCBI) Sequence Read Archive (SRA), being associated with the following BioProjects; PRJNA578806 (*Eucalyptus albens* and *Eucalyptus sideroxylon*) [9], PRJNA743927 (*Oryza australiensis*) [16], PRJNA749614 (*Puccinia striiformis* f. sp. *tritici*) [11], PRJNA725323 (*Puccinia triticana*) [12]. Various other *Eucalyptus* species and *Acacia* species are being made available under BioProjects PRJNA509734 and PRJNA510265 respectively. Supporting publications and data of other genera are soon to follow.

## Supporting information

S1 File Step-by-step protocol, also available on Protocols.io. (PDF)

S2 File Supplemental repository of custom dual index primers, program files for PerkinElmer automated workstations, excel files for analysing microplate readings and a comparison of protocol prices. (ZIP)
