# Supplementary material for: Cost-conscious generation of multiplexed short-read DNA libraries for whole-genome sequencing
Source: PLoS One. 2023 Jan 27;18(1):e0280004. doi: 10.1371/journal.pone.0280004 (PMC9882895; doi:10.1371/journal.pone.0280004)
Supplement: S1 File — (PDF) [file pone.0280004.s001.pdf]

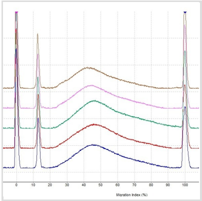

Version 2

Jul 17, 2022

Cost-conscious generation of multiplexed short-read DNA libraries for whole-genome sequencing V.2

Ashley Jones[1], David Stanley[1,2], Scott Ferguson[1], Benjamin Schwessinger[1], Justin Borevitz[1], Norman Warthmann[1,3]

[1]Research School of Biology, Australian National University. Canberra, ACT Australia;

[2]Diversity Arrays Technology, Bruce, ACT, Australia;

[3]Plant Breeding and Genetics Laboratory (PBGL), Joint FAO/IAEA Center of Nuclear Techniques in Food and Agriculture, International Atomic Energy Agency (IAEA), IAEA Laboratories, 2444 Seibersdorf, Austria

1 Works for me   Share

dx.doi.org/10.17504/protocols.io.14egnx27zl5d/v2

EBL_ANU   Team Schwessinger

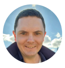

Ashley Jones
Australian National University

ABSTRACT

Massively parallel, second-generation short-read DNA sequencing has become an integral tool in biology for genomic studies. Offering highly accurate base-pair resolution at the most competitive price, the technology has become widespread. However, high-throughput generation of multiplexed DNA libraries can be costly and cumbersome. Here, we present a cost-conscious protocol for generating multiplexed short-read DNA libraries using a bead-linked transposome from Illumina. By preparing libraries in high-throughput with small reaction volumes that use 1/50th the amount of transposome compared to Illumina DNA Prep tagmentation protocols, the cost per library can be substantially reduced, by approximately 1/20th. Furthermore, we optimised the protocol to minimise magnetic bead-based clean-ups between steps, further reducing cost, time and DNA input requirements. By developing our own dual index primers to multiplex nine 96-well microplates, up to 864 samples can be placed on a single flow cell. This enables efficient usage of large-scale sequencing platforms, such as the Illumina NovaSeq 6000, which offers up to three terabases of sequencing per S4 flow cell.

DOI

dx.doi.org/10.17504/protocols.io.14egnx27zl5d/v2

Citation: Ashley Jones, David Stanley, Scott Ferguson, Benjamin Schwessinger, Justin Borevitz, Norman Warthmann Cost-conscious generation of multiplexed short-read DNA libraries for whole-genome sequencing https://dx.doi.org/10.17504/protocols.io.14egnx27zl5d/v2

This is an open access protocol distributed under the terms of the **Creative Commons Attribution License**

PROTOCOL CITATION

Ashley Jones, David Stanley, Scott Ferguson, Benjamin Schwessinger, Justin Borevitz, Norman Warthmann 2022. Cost-conscious generation of multiplexed short-read DNA libraries for whole-genome sequencing. **protocols.io**
https://dx.doi.org/10.17504/protocols.io.14egnx27zl5d/v2
Version created by Ashley Jones

KEYWORDS

Illumina, tagmentation, short-reads, multiplexed libraries, DNA sequencing

LICENSE

This is an open access protocol distributed under the terms of the Creative Commons Attribution License, which permits unrestricted use, distribution, and reproduction in any medium, provided the original author and source are credited

CREATED

Feb 04, 2021

LAST MODIFIED

Jul 17, 2022

PROTOCOL INTEGER ID

46929

GUIDELINES

This protocol is designed for Illumina short-read sequencing and requires previously purified DNA. The DNA will be diluted, tagmented and later amplified, thus only a small input is needed, approximately 3 ng. To aid conducting this protocol, program files for automated workstations by PerkinElmer have been provided in the supplemental with descriptions and compatible hardware. Helpful excel sheets to create standard curves for DNA quantification and dilution strategies are also provided. Lastly, custom dual index primers are provided to multiplex nine unique 96-well microplates, enabling multiplexing up to 864 samples on a single sequencing run.

supplemental.zip

MATERIALS TEXT

Consumables

- Illumina DNA Prep, (M) Tagmentation, 24 sample kit (Illumina 20060060, originally 20018704) or 96 sample kit (20060059, originally 20018705). Note: these products were previously known as Nextera DNA Flex Library Prep.

Citation: Ashley Jones, David Stanley, Scott Ferguson, Benjamin Schwessinger, Justin Borevitz, Norman Warthmann Cost-conscious generation of multiplexed short-read DNA libraries for whole-genome sequencing
https://dx.doi.org/10.17504/protocols.io.14egnx27zl5d/v2

This is an open access protocol distributed under the terms of the **Creative Commons Attribution License**

- Custom dual index primers (see supplemental). Alternatively, IDT for Illumina DNA/RNA Unique Dual Indexes Tagmentation (96 Indexes, 96 Samples), Set A (Illumina 20027213), Set B (20027214), Set C (20027215), or Set D (20027216).
- Custom tagmentation buffer (see 'before start', 2x TMP: 20 mM Tris-HCl pH 8, 10 mM $MgCl_2$, 16% PEG 8,000).
- Tris-HCl pH 8 (e.g. UltraPure 1M Tris-HCl, pH 8.0, Thermo Fisher Scientific 15568025).
- Magnesium chloride (e.g. 1 M $MgCl_2$, Thermo Fisher Scientific AM9530G).
- Poly(ethylene glycol) 8,000 (e.g. PEG 8,000 BioUltra, Merck 89510-1KG-F).
- Nuclease-free water (e.g. UltraPure DNase/RNase-Free Distilled Water, Thermo Fisher Scientific 10977015).
- dsDNA Quantification High Sensitivity Kit (for microplate reader) (BioDynami 40540H). The broad range assay kit may be required depending on the concentration of samples (BioDynami 40540B). Alternatives: Quant-iT dsDNA Assay Kit, high sensitivity (HS) and broad range (BR) (Thermo Fisher Scientific Q33120 and Q33130 respectively).
- dsDNA Quantification High Sensitivity Kit (BioDynami 40042L) (required if doing protocol in low-throughput). The broad range assay kit may be required depending on the concentration of samples (BioDynami 40041L). Alternatives: Qubit dsDNA Quantification Assay Kits, high sensitivity (HS) and broad range (BR) (Thermo Fisher Scientific Q32854 and Q32853 respectively).
- Qubit assay tubes (Thermo Fisher Scientific Q32856).
- Q5 High-Fidelity DNA Polymerase (New England BioLabs M0491L).
- 10 mM dNTPs (New England BioLabs N0447).
- 70% ethanol (analytical grade or higher).
- AMPure XP Reagent, SPRI paramagnetic beads (Beckman Coulter A63881) (or equivalent beads). May not be needed, depends on clean-up strategy.
- 96-well half skirt PCR microplate (e.g. Axygen Scientific PCR-96M2-HS-C).
- 96-well microplate suitable for fluorescent readings, e.g. Microplate, 96 well, PS, half area, black, med. binding (Greiner Bio-One 675076), or alternatively, 96-well PCR microplate white, Roche 480 light cycler (Axygen Scientific PCR-96-LC480-W-NF).
- 384-well microplate (e.g. 4titude FrameStar 4ti-0384/B).
- PCR-grade adhesive seals for microplates (e.g. Thermo Fisher Scientific AB0558).
- 2 mL SC micro tube PCR-PT (Starstedt 72694.406) (or equivalent, for PerkinElmer automated workstations)
- 1.5 mL microcentrifuge tubes (e.g. Eppendorf tubes).
- 15 mL and/or 50 mL centrifuge tubes (e.g. Falcon tubes).

Equipment

- FLUOstar Omega fluorescent microplate reader (BMG Labtech), or equivalent, e.g. Infinite M1000 PRO (Tecan).
- PCR machine for 96-well microplate.
- PCR machine for 384-well microplate, or a microplate heat block.
- Magnetic rack for 1.5 mL microcentrifuge tubes.
- Qubit Fluorometer (Thermo Fisher Scientific).
- (Optional) JANUS automated workstation, such as NGS express or JANUS G3, including relevant accessories; reagent reservoirs, plate adaptor support tiles, 25 and 175 µL filtered tips (PerkinElmer).

Citation: Ashley Jones, David Stanley, Scott Ferguson, Benjamin Schwessinger, Justin Borevitz, Norman Warthmann Cost-conscious generation of multiplexed short-read DNA libraries for whole-genome sequencing https://dx.doi.org/10.17504/protocols.io.14egnx27zl5d/v2

This is an open access protocol distributed under the terms of the **Creative Commons Attribution License**

- (Optional) PippinHT (Sage Science), including 2% agarose casette and reagents.
- (Optional) High sensitive electrophoresis system, such as LabChip GX devices (PerkinElmer) or Bioanalyzer/TapeStation devices (Agilent Technologies).

BEFORE STARTING

In this protocol, the transposome being used will be diluted 1/50, therefore up to 50 times more tagmentation reaction buffer is required. Prepare the following custom tagmentation buffer in adavance (example ordering details are in the materials section).

- 2x TMP: 20 mM Tris-HCl pH 8, 10 mM $MgCl_2$, 16% PEG 8,000.
- Final concentration in tagmentation reaction is 10 mM Tris-HCl, 5 mM $MgCl_2$, 8% PEG 8,000.
- Filter sterilise the solution and store at 4°C (fridge) for up to one year.

A	B	C	D	E
**Reagent**	**Target concentration**	**MW**	**Stock concentration**	**From stock**
Tris-HCl (pH 8)	20 mM	121.14	1 M	20 µL
MgCl2	10 mM	95.21	1 M	10 µL
PEG 8,000	16%	8,000	25%	640 µL
Nuclease-free water	NA	NA	NA	330 µL
-	-	-	-	**Total: 1 mL**

DNA quantification

1 Quantify the DNA samples. For a low-throughput number of samples, quantify with a Qubit Fluorometer according to the manufacturer's instructions (Thermo Fisher Scientific). The following describes quantification in high-throughput (96-well microplates) by reading on a fluorescent microplate reader.

2 To measure a 96-well microplate of DNA samples with a dsDNA quantification high sensitivity kit (for microplate reader) (BioDynami or Thermo Fisher Scientific), ensure concentrations are ≤ 33 ng/µL. For instance, first quantify a few samples on a Qubit Fluorometer. Highly concentrated microplates of DNA can be diluted 1/10 before starting (e.g. 5 µL of DNA added to 45 µL nuclease-free water in a new 96-well microplate).

- *dsDNA quantification high sensitivity kits have a quantification range of 0.2 to 100 ng. This protocol will quantify 3 µL of DNA at a concentration of ≤ 33 ng/µL.*

Citation: Ashley Jones, David Stanley, Scott Ferguson, Benjamin Schwessinger, Justin Borevitz, Norman Warthmann Cost-conscious generation of multiplexed short-read DNA libraries for whole-genome sequencing https://dx.doi.org/10.17504/protocols.io.14egnx27zl5d/v2

This is an open access protocol distributed under the terms of the **Creative Commons Attribution License**

- *Highly concentrated samples are difficult to measure on microplate readers. Reducing gain on fluorescent microplate reader to 80% can help but will be at the cost of accuracy.*

3 Create a quantification working solution by mixing dsDNA HS dye/reagent 1:200 with dsDNA buffer. Each reaction will require 97 µL of the working solution and 3 µL of DNA sample (or standard) will be added later (100 µL total volume). For a whole 96-well microplate, create enough buffer for 96 samples, 32 standards and 22 extra samples dead volume as follows:

A	B	C	D	E	F	G
**Number of plates**	**Samples**	**Standards**	**Dead volume**	**Total**	**dsDNA buffer**	**dsDNA HS reagent**
1	96	32	22	150	14,477 µL	73 µL
2	192	64	44	300	28,954 µL	146 µL

- *This reaction setup successfully uses half reactions compared to manufacturer protocols, i.e. 100 µL per sample instead of 200 µL.*
- *JANUS automated workstations (PerkinElmer) generally require at least 1 mL of dead volume in large reservoirs. If pipetting manually with a multi-channel pipette, the dead volume can be reduced, to approximately 12 samples per 96-well microplate.*

4 For each 96-well microplate of DNA samples, prepare two microplates suitable for a fluorescent microplate reader. As standards will need to be read in addition to samples, the orginal 96-well microplate of samples will need to be split across two microplates (label as microplate A and B, or 1-48 and 49-96 etc).

Example microplates are listed in the materials. These plates can be washed, stored and re-used for future quantifications.

5 Transfer 97 µL of quantification working solution into the first two columns of each microplate (16 wells each microplate). This will be used for 8 standards, done in duplicate on each microplate.

Citation: Ashley Jones, David Stanley, Scott Ferguson, Benjamin Schwessinger, Justin Borevitz, Norman Warthmann Cost-conscious generation of multiplexed short-read DNA libraries for whole-genome sequencing https://dx.doi.org/10.17504/protocols.io.14egnx27zl5d/v2

This is an open access protocol distributed under the terms of the **Creative Commons Attribution License**

Having standards on each microplate is necessary for accuracy when generating standard curves later.

6 Transfer 97 µL of quantification working solution into the microplates for half of the the DNA samples. For example, filling columns 4-9 on microplate A, and 5-10 on microplate B, as follows:

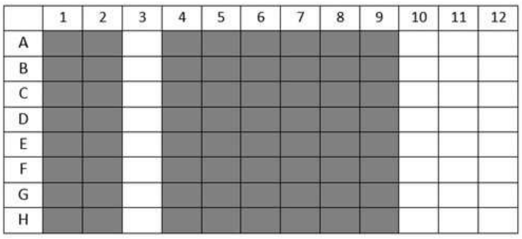

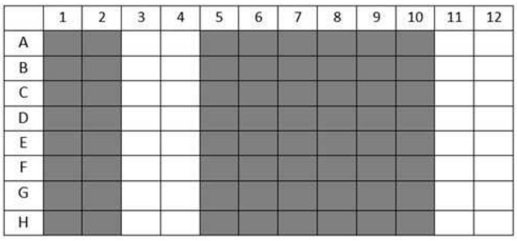

It is good practice to be able to distinguish the two microplates from one another to avoid confusion and/or mistakes. Here, microplate A will have columns 1-2 as standards, column 3 as blank, columns 4-9 as DNA samples. Microplate B will have two columns as blank between the standards and DNA samples. For instance: columns 1-2 as standards, columns 3-4 as blank, columns 5-10 as DNA samples.

7 Add 3 µL of each standard to the microplate columns dedicated to standards. Perform this in duplicate.

Add standards in increasing concentration: 0, 0.5, 1, 2, 4, 6, 8 and 10 ng/µL. Then duplicate in the next column.

8 Add 3 µL of each DNA sample to the microplate columns dedicated to samples.

9 Seal the microplate with adhesive film, vortex and briefly spin down. Incubate for 5 min.

[illegible]

Citation: Ashley Jones, David Stanley, Scott Ferguson, Benjamin Schwessinger, Justin Borevitz, Norman Warthmann Cost-conscious generation of multiplexed short-read DNA libraries for whole-genome sequencing https://dx.doi.org/10.17504/protocols.io.14egnx27zl5d/v2

This is an open access protocol distributed under the terms of the **Creative Commons Attribution License**

The fluorescence signal is stable for approximately 3 h at room temperature, when protected from light.

10 Measure fluorescence using a microplate reader, using standard fluorescein wavelengths, for example excitation/emission at ~480/530 nm.

- *For dsDNA Quantification Kits (for microplate reader) (BioDynami), the maximal excitation/emission are 495/540 nm (high sensitivity kit) and 485/545 nm (broad range kit). Standard fluorescent excitation/emission at ~480/530 nm is appropriate (for both high sensitivity and broad range kits).*
- *For Quant-iT dsDNA Assay Kits (Thermo Fisher Scientific), the maximal excitation/emission are ~502/523 nm (high sensitivity kit) and ~510/527 nm (broad range kits). Standard fluorescent excitation/emission at ~480/530 nm is appropriate (for both high sensitivity and broad range kits).*
- *For a FLUOstar Omega (BMG Labtech), select whole microplate (not single well) when setting gain. A gain of 80% has been suitable for this workflow.*
- *For an Infinite M1000 PRO (Tecan), use a bandwidth of 5 nm, top mode, flashes: 100 Hz x10, Z-position: 20,000 µm, gain can be optimal or set a manual gain (e.g. 96%).*
- *Lower the gain if sample concentrations vary substantially, as fluorescence may be too high. However, this will reduce overall accuracy of the quantification.*
- *Ensure the correct microplate settings are entered.*

11 Utilising the known concentration of standards included on the microplate, create a standard curve to determine DNA concentration of the other samples.

- *Plot the quantity of standards against fluorescence and fit a straight line to the points.*
- *Example files have been provided in the supplemental.*

DNA dilution

12 Dilute all DNA samples to 1-2 ng/µL in a volume of approximately 25 µL. For example, aim for 1.5 ng/µL for each sample. First transfer nuclease-free water to a new 96-well PCR microplate

Citation: Ashley Jones, David Stanley, Scott Ferguson, Benjamin Schwessinger, Justin Borevitz, Norman Warthmann Cost-conscious generation of multiplexed short-read DNA libraries for whole-genome sequencing
https://dx.doi.org/10.17504/protocols.io.14egnx27zl5d/v2

This is an open access protocol distributed under the terms of the **Creative Commons Attribution License**

and then add the appropriate amount of DNA.

- *For JANUS automated workstations (PerkinElmer), files have been provided in the supplemental to help calculate the dilutions.*
- *When using a JANUS automated workstation, note that the most suitable tip size is 25 µL, however highly concentrated samples may need a larger volume of water to be transferred than 25 µL.*

13 Ensure the diluted samples are mixed and briefly spin down if necessary. Can be sealed with adhesive film and stored at -20°C until ready for tagmentation.

14 If necessary, the DNA can be quantified and diluted again as previously described.

The Illumina Bead-Linked Transposome (BLT) alleviates the need for further diluting and quantifying, being able to accomodate a wide concentration of samples.

Tagmentation

15 Pre-heat a PCR machine or heat block at 53°C suitable for a 384-well microplate. Will be used for the tagmentation reaction later.

Many protocols use 55°C for the tragmentation reaction, however, if there is a chance of overheating, the transposome can denature. Lower temperature of 53°C has been suitable for this protocol.

16 Resuspend the Bead-Linked Transposome (BLT), thoroughly by flick-mixing and brief vortexing.

BLT is from the Illumina DNA Prep Kit.

Citation: Ashley Jones, David Stanley, Scott Ferguson, Benjamin Schwessinger, Justin Borevitz, Norman Warthmann Cost-conscious generation of multiplexed short-read DNA libraries for whole-genome sequencing
https://dx.doi.org/10.17504/protocols.io.14egnx27zl5d/v2

This is an open access protocol distributed under the terms of the **Creative Commons Attribution License**

17 Prepare a tagmentation master mix in a 1.5 mL microcentrifuge tube. For an entire 96-well microplate of samples, prepare for 102 samples (i.e. a dead volume of 6 samples), as follows.

A	B	C
**Reagent**	**Per sample**	**For 102 samples**
2x TMP (custom buffer)	3 µL	306 µL
Transposome (BLT)	0.2 µL	20.4 µL
DNA (at 1-2 ng/µL)	2.8 µL	-
**TOTAL**	**6 µL**	**326.4 µL**
**Aliquot per sample**	-	**3.2 µL**

- *Requires the custom tagmentation buffer detailed previously.*
- *Uses 1/50th the amount of BLT compared to Illumina protocols (0.2 µL instead of 10 µL).*
- *DNA concentration can be variable, however 1-2 ng/µL is ideal for this protocol.*

18 Transfer 3.2 µL of the tagmentation master mix into 96-wells of a 384-well microplate (for example, the first quadrant in each set of four wells).

- *Perform tagmentation in 384-well microplate due to the low reaction volume and to enable aspiration with automated workstations.*
- *Depending on labortaory set-up and equipment being used, a 96-well microplate can also be suitable. For example, in low-throughput experiments, the same 96-well microplate can be used for tagmentation and PCR.*
- *Left-over tagmentation master mix can be stored at 4°C for later use, but storage life is limited.*

19 Transfer 2.8 µL of DNA (at 1-2 ng/µL) to each of the 96-wells containing tagmentation master mix. Gently pipette mix after the transfer.

Citation: Ashley Jones, David Stanley, Scott Ferguson, Benjamin Schwessinger, Justin Borevitz, Norman Warthmann Cost-conscious generation of multiplexed short-read DNA libraries for whole-genome sequencing
https://dx.doi.org/10.17504/protocols.io.14egnx27zl5d/v2

This is an open access protocol distributed under the terms of the **Creative Commons Attribution License**

20 Seal the microplate with adhesive film (PCR-grade), lightly vortex, keeping the liquid in the bottom of the well to avoid centrifuging.

Gently centrifuge only if necessary. Bead-Linked Transposome (BLT) will pellet easily.

21 Incubate microplate at 53°C for 30 min, mixing at 300-450 rpm if possible. If not in a shaker, mix by lightly vortexing after 15 min then put back for another 15 min.

Start preparing the PCR setup during this time.

22 After incubation, remove microplate from heat block and keep at room temperature until PCR.

- *The tagmentation reaction will go to completion, inserting all available adapters (therefore doesn't require a stopping solution).*
- *No clean-up of this reaction is necessary, due to the low volume of BLT used and custom tagmentation buffer developed.*

PCR for enrichment and barcoding

23 Prepare a PCR master mix without the index primers and the tagmentation reaction (the template). For an entire 96-well microplate of samples, prepare for 102 samples (i.e. a dead volume of 6 samples). Alternatively, if using an automated workstation e.g. JANUS G3 (PerkinElmer), prepare for 28 samples in quadruplicate (i.e. 24 samples and 4 extra as dead volume), in four separate 1.5 mL microcentrifuge tubes. Prepare as follows.

Citation: Ashley Jones, David Stanley, Scott Ferguson, Benjamin Schwessinger, Justin Borevitz, Norman Warthmann Cost-conscious generation of multiplexed short-read DNA libraries for whole-genome sequencing https://dx.doi.org/10.17504/protocols.io.14egnx27zl5d/v2

This is an open access protocol distributed under the terms of the **Creative Commons Attribution License**

A	B	C	D
**Reagent**	**Per sample**	**For 102**	**(For 28) x4**
Nuclease-free water	28.15 µL	2,871.3 µL	788.2 µL
5x Q5 reaction buffer	7.5 µL	765 µL	210 µL
10 mM dNTPs	1 µL	102 µL	28 µL
Q5 High-Fidelity DNA Polymerase	0.35 µL	35.7 µL	9.8 µL
2.5 µM i5 index primer	4 µL	-	-
2.5 µM i7 index primer	4 µL	-	-
Tagmentation reaction	5 µL	-	-
**TOTAL**	**50 µL**	**3,774 µL**	**1,036 µL**
**Aliquot per sample**	-	**37 µL**	**37 µL**

- *PCR will be performed in a 96-well microplate; tagmentation reaction will be transferred from a 384-well to 96-well microplate.*

- *Less than 1x of the Q5 reaction buffer will be present in each reaction. This has been calculated based on current salt concentrations in the non-cleaned tagmentation reaction.*

Key notes on PCR reaction conditions:

- *Key to PCR success was double volume (50 µL), no DMF from tagmentation reaction (but BLT may come with small amount of DMF), no extra glycerol and no 5x Q5 GC Enhancer (has both DMSO and glycerol).*
- *There is PEG carry-over from tagmentation reaction (8% in tagmentation reaction, 0.8% in PCR).*
- *Optimal Mg^{2+} for Q5 polymerase is 2 mM.*
- *5 µL of tagmentation reaction (5 mM $MgCl_2$) in 50 µL PCR makes $MgCl_2$ 0.5 mM*
- *NEB 5x Q5 reaction buffer is 10 mM $MgCl_2$. Adding 7.5 µL in 50 µL PCR is 1.5 mM $MgCl_2$*
- *Final $MgCl_2$ is now at optimal ~2 mM*
- *Other additives in 5x Q5 reaction buffer are not known.*
- *Amount of Q5 polymerase has been reduced (NEB recommends 0.5 µL Q5 per 50 µL volume).*
- *Final primer concentration is 0.2 µM each (PCR typically uses 0.1-1 µM each primer).*

Citation: Ashley Jones, David Stanley, Scott Ferguson, Benjamin Schwessinger, Justin Borevitz, Norman Warthmann Cost-conscious generation of multiplexed short-read DNA libraries for whole-genome sequencing https://dx.doi.org/10.17504/protocols.io.14egnx27zl5d/v2

This is an open access protocol distributed under the terms of the **Creative Commons Attribution License**

24 Transfer 37 µL of PCR master mix to each well of a 96-well PCR microplate.

25 Add 4 µL of a 2.5 µM i5 index primer and 4 µL of a 2.5 µM i7 index primer to each sample. Ensure each well has a unique combination of dual index primers.

- *Eight unique i5 index primers will need to be arrayed horizontally, across the eight rows of the microplate (one i5 index for a whole row). Twelve unique i7 index primers will need to be arrayed vertically, down the twelve columns of the microplate (one i7 index for a whole column).*
- *Dual index primer sequences and recommended combinations are provided in the supplemental.*
- *This step can be prone to human error. Ensure tubes are labelled and microplate orientations are accounted for (e.g. include blanks in decipherable locations per microplate). Using an automated workstation such as a JANUS G3 (PerkinElmer) is highly recommended.*

26 Transfer 5 µL of tagmentation reaction (template) from the 384-well microplate to the 96-well microplate containing the PCR master mix aliquots.

27 Seal microplate with adhesive film (PCR-grade), vortex and briefly centrifuge (avoid pelleting the beads).

28 Perform PCR as follows:

72°C for 3 min
98°C for 3 min

12-16 cycles of:
- 98°C for 45 s
- 62°C for 30 s
- 72°C for 2 min

72°C for 1 min
Hold at ≤ 10°C

Citation: Ashley Jones, David Stanley, Scott Ferguson, Benjamin Schwessinger, Justin Borevitz, Norman Warthmann Cost-conscious generation of multiplexed short-read DNA libraries for whole-genome sequencing https://dx.doi.org/10.17504/protocols.io.14egnx27zl5d/v2

This is an open access protocol distributed under the terms of the **Creative Commons Attribution License**

Avoid high number of PCR cycles to limit duplicate fragments and formation of hetero-duplexes. Currently we have been regularly using 16 cycles however believe it can now be reduced.

29 Proceed to pooling or store at -20°C.

Pool libraries

30 Quantify the DNA libraries as previously described, using a dsDNA quantification high sensitivity kit for microplate reader (BioDynami or Thermo Fisher Scientific), or a Qubit Fluorometer for low-throughput samples.

- *Optional, but this helps provide a more uniform pooling of samples. Pooling samples based on concentration asssumes samples have a similar average insert size; true molarity may vary.*
- *To measure molarity, use a high sensitive automated electrophoresis system, such as LabChip GX devices (PerkinElmer) or Bioanalyzer/TapeStation devices (Agilent Technologies).*

31 Add 40 µL of nuclease-free water into 1.5 mL microcentrifuge tube to start the pool.

Optional, but this is ideal for automated workstations.

32 Transfer an equal amount of each library (based on concentration, molarity or use a set volume e.g. 10 µL) from each well of the microplate into the same 1.5 mL microcentrifuge tube. This can be performed on an automated workstation (e.g. JANUS G3 by PerkinElmer), providing a csv file with volumes to transfer for each sample.

10 µL x 96 samples + initial 40 µL = 1,000 µL.

Citation: Ashley Jones, David Stanley, Scott Ferguson, Benjamin Schwessinger, Justin Borevitz, Norman Warthmann Cost-conscious generation of multiplexed short-read DNA libraries for whole-genome sequencing
https://dx.doi.org/10.17504/protocols.io.14egnx27zl5d/v2

This is an open access protocol distributed under the terms of the **Creative Commons Attribution License**

33 Place the tube on a magnetic rack for 5 min, or until all beads pellet.

34 Keeping the tube on the magnetic rack, transfer the supernatant to a new tube, without disturbing the beads. The beads can be discarded.

- *These steps are only necessary when using the bead-linked transposome (BLT). The amplified library is free in solution and not attached to the beads.*
- *It is good practice to save an aliquot (e.g. 10 µL) of the pool for QC analysis on a high sensitive automated electrophoresis systems, such as LabChip GX devices (PerkinElmer) or Bioanalyzer/TapeStation devices (Agilent Technologies).*

Clean-up and size selection (AMPure XP)

35 The pooled library can now undergo clean-up and size selection, which is flexible and multiple methods exist. For example, use AMPure XP Reagent (SPRI paramagnetic beads) according to the manufacturer's instructions (Beckman Coulter). Typically, 0.5-1x of the beads are addded relative to the total volume of the pool.

As the beads provided in the Illumina DNA prep tagmentation sample kit havent been consumed by the protocol, they could be used here following the manufacturer's instructions, by adjusting for the different volume.

36 It may be necessary to repeat the clean-up to remove all primers and short fragments from the pool. For example a second 0.6x AMPure XP size selection according to the manufacturer's inctructions (Beckman Coulter).

37 Alternatively (or additionally), the library can udergo clean-up and size selection using a PippinHT (Sage Science), as detailed below.

Clean-up and size selection (PippinHT)

38 Prior to PippinHT size selection, the library may need to be concentrated, which can be done with AMPure XP beads (or equivalent) according to the manufacturer's inctructions (Beckman Coulter).

Alternatively, to concentrate large pools of library, use a MinElute PCR Purification Kit

Citation: Ashley Jones, David Stanley, Scott Ferguson, Benjamin Schwessinger, Justin Borevitz, Norman Warthmann Cost-conscious generation of multiplexed short-read DNA libraries for whole-genome sequencing https://dx.doi.org/10.17504/protocols.io.14egnx27zl5d/v2

This is an open access protocol distributed under the terms of the **Creative Commons Attribution License**

Alternatively, to concentrate large pools of library, use a MinElute PCR Purification Kit (Qiagen 28004), according to the manufacturer's inctructions.

39 To perform a stringent size selection, a PippinHT (Sage Science) can be used. This will remove undesirable shorter fragments that will have overlapping sequencing on paired-end platforms. Using a PippinHT, perform an automatic size selection and gel purification, following the manufacturer’s instructions (Sage Science). A 2% agarose casette and reagents are ideal for Illumina short-read libraries. A size selection of 430 - 600 bp is ideal for Illumina 150 bp paired-end sequencing, but will depend on sequencing platform and the quantity of pooled libary required.

- *The PippinHT has 12 lanes, markers can be internal (recommended) or external. Each lane requires 20 µL of DNA combined with 5 µL of marker. The manufacturer recommends a maximum 1.5 µg per lane. We have often overloaded to 3 µg per lane with minimal to no consequence to the size selection.*
- *Note the dual index primers and tagmentation adapters add 136 bp to the amplified library length, therefore library sizes approximately 436 bp are ideal for 150 bp paired-end sequencing.*
- *The higher the stringency of the size selection, the lower the recovery of library. Have a wider size selection range to recover more library (e.g. 350 - 1,000 bp).*

40 After PippinHT size selection, the library can be cleaned and concentrated with AMPure XP beads (or equivalent) according to the manufacturer's inctructions (Beckman Coulter).

Preparation for sequencing

41 Perform a quality check analysis of the final library pool on a high sensitive automated electrophoresis system, such as LabChip GX devices (PerkinElmer) or Bioanalyzer/TapeStation devices (Agilent Technologies).

- *This can be done by your local sequencing facility.*
- *It is good practice to include an aliquot from the raw (unclean) pool for comparison.*

Citation: Ashley Jones, David Stanley, Scott Ferguson, Benjamin Schwessinger, Justin Borevitz, Norman Warthmann Cost-conscious generation of multiplexed short-read DNA libraries for whole-genome sequencing
https://dx.doi.org/10.17504/protocols.io.14egnx27zl5d/v2

This is an open access protocol distributed under the terms of the **Creative Commons Attribution License**

42 Ensure the library looks as expected, relative to size selection and sequencing strategy. For Illumina NovaSeq 6000 150 bp paired-end sequencing, a library 400-600 bp is ideal. Estimate the average fragment length.

- *A double peak is indicative of over-amplification; primers may become limited, PCR products may anneal, forming/amplifying fragments that are twice as large (such as hetero-duplexes). Quantification and calculation of molarity will likely not be precise.*
- *A stretched peak, similar to a double peak, may form if the electrophoresis system is overloaded. This is referred to as 'bird nesting'; the DNA fragments get intertwined and appear longer. This is not necessarily an issue; denaturation during sequencing bridge PCR will eliminate this tangle.*
- *If the library size is very large (e.g. greater than 1 kb), there is not enough transposition occuring in the tagmentation reaction. Something may be inhibiting the tagmentation reaction if the DNA is highly contaminated. Alternatively, DNA input may have been too high; check the concentration and reduce the DNA input if necessary.*

43 Quantify the library at least twice with a Qubit Fluorometer, using a Qubit dsDNA high sensitivity assay kit. Determine the average of the readings.

44 Calculate molarity; nM = (ng/µL x 10^6) / (660 g/mol x average fragment size bp).

Although high sensitive automated electrophoresis systems will provide a molarity calculation, we have found molarity based on Qubit values and an average fragment length the most accurate.

45 Dilute the library to a suitable molarity for sequencing. Store at -20°C until ready for sequencing.

Most sequencing facilities will require libraries ~2-10 nM for Illumina platforms. Check with local sequencing provider before diluting.

Citation: Ashley Jones, David Stanley, Scott Ferguson, Benjamin Schwessinger, Justin Borevitz, Norman Warthmann Cost-conscious generation of multiplexed short-read DNA libraries for whole-genome sequencing https://dx.doi.org/10.17504/protocols.io.14egnx27zl5d/v2

This is an open access protocol distributed under the terms of the **Creative Commons Attribution License**

46 Send for sequencing, on ice, cold packs or dry ice depending on the distance to the closest sequencing facility.

> *The Illumina NovaSeq 6000 is recommended, using a flowcell with 300 cycles (150 bp paired-end sequencing).*

Citation: Ashley Jones, David Stanley, Scott Ferguson, Benjamin Schwessinger, Justin Borevitz, Norman Warthmann Cost-conscious generation of multiplexed short-read DNA libraries for whole-genome sequencing https://dx.doi.org/10.17504/protocols.io.14egnx27zl5d/v2

This is an open access protocol distributed under the terms of the **Creative Commons Attribution License**
