# Supplementary material for: Cost-conscious generation of multiplexed short-read DNA libraries for whole-genome sequencing
Source: PLoS One. 2023 Jan 27;18(1):e0280004. doi: 10.1371/journal.pone.0280004 (PMC9882895; doi:10.1371/journal.pone.0280004)
Supplement: S2 File — (ZIP) [file pone.0280004.s002.zip › supplemental/program_files_PerkinElmer_workstations/PerkinElmer JANUS G3/program_descriptions_JANUS_G3.pdf]

Program files for JANUS G3 automated workstation (PerkinElmer)

Cost-conscious generation of multiplexed short-read DNA libraries for whole-genome sequencing

Jones *et al.* (2022)

These programs will depend on specific consumables and will pipette variable volumes according to a csv file. Users will need ensure they are using compatible consumables (or alter the program) and adjust the pipetting csv file to your specifications. Users must prepare master mixes in advance. If pipetting out of the trough/reservoir, at least 1 mL of dead volume will be required. If pipetting from 2 mL screw cap or 1.5 mL microcentrifuge tubes (within the 24-rack), cater for approximately 40 µL dead volume.

1. Quantification

Program and file:

- quantification_transfer_two_plates_greiner_black.MPT
- quantification_transfer_two_plates_greiner_black.csv

Microplates:

- DNA source: 96-well half skirt PCR microplate (Axygen Scientific PCR-96M2-HS-C)
- Quantification: Microplate, 96 well, PS, half area, black, med. Binding, 10 pcs/bag. (Greiner Bio-One 675076).

Microcentrifuge tubes:

- Qubit standards are equivalent to 2 mL SC micro tube PCR-PT (Starstedt 72694.406).

Pipette tips:

- Master mix aliquoted with 175 µL filtered non-conductive (PerkinElmer).
- All others are 25 µL filtered non-conductive (PerkinElmer).

For fluorescent measurement in a microplate reader. Adds 97 µL buffer/dye mix to each well. Adds 3 µL of the sample, and it is advised to have the series of standards on every microplate. Destination consumable for quantification is the 96-well black microplate (Greiner Bio-One). Master mix in the reservoir is aliquoted into two of these microplates with 175 µL tips. Standards from 2 mL tube are transferred to each of these microplates. DNA from 96-well half skirt PCR microplate (Axygen Scientific) is then transferred to these microplates, split half /half. A user-supplied csv dictates volumes and destinations.

Note: A one-column difference between microplates is designed to make the microplates visually distinct:

Xa	1	2	3	4	5	6	7	8	9	10	11	12
A												
B												
C												
D												
E												
F												
G												
H												

Xb	1	2	3	4	5	6	7	8	9	10	11	12
A												
B												
C												
D												
E												
F												
G												
H												

2. Dilution

Program and file:

- dilution_new_plate_user_supply_file.MPT
- dilution_new_plate_user_supply_file_example.csv

Microplates:

- Both: 96-well half skirt PCR microplate (Axygen Scientific PCR-96M2-HS-C).

Pipette tips:

- 25 µL, filtered non-conductive (PerkinElmer).

Dilutes to a desired concentration, <u>cannot</u> add more than 25 µL due to tip capacity. Best to avoid pipetting less than 2 µL; dilute manually before, or do several iterations. Program transfers water from reservoir to a new 96-well half skirt PCR microplate. Transfers stock DNA (in a different 96-well half skirt PCR microplate) to the new microplate prepared, then mixes the DNA and water. A user-supplied csv dictates the volume of water and DNA.

3. Tagmentation

Programs and file:

- tagmentation_from_96well_to_384well_nonconductive.MPT
- tagmentation_from_96well_to_384well_conductive.MPT
- tagmentation_from_96well_to_384well.csv

Microplates:

- DNA source: 96-well half skirt PCR microplate (Axygen Scientific PCR-96M2-HS-C).
- Tagmentation: 384-well microplate (4titude FrameStar 4ti-0384/B),

Microcentrifuge tubes:

- 1.5 mL microcentrifuge tube (for tagmentation master mix).

Pipette tips:

- Master mix aliquoted with 25 µL filtered conductive or non-conductive tips, both options provided (PerkinElmer).
- All others are 25 µL filtered non-conductive (PerkinElmer).

Tagmentation master mix in a 1 .5 mL microcentrifuge tube is aliquoted into the 384-well microplate (4titude FrameStar). Stock DNA in a 96-well half skirt PCR microplate (Axygen Scientific) is then transferred into the fortitude 384-well microplate. A user-supplied csv dictates the volume of master mix and DNA.

4. PCR

Program and file:

- pcr_50ul_oligo_array_tagmentation_transfer_384_96.MPT
- pcr_50ul_oligo_array_tagmentation_transfer_384_96.csv

Microplates:

- Tagmentation: 384-well microplate (4titude FrameStar 4ti-0384/B)
- PCR: 96-well half skirt PCR microplate (Axygen Scientific PCR-96M2-HS-C).

Microcentrifuge tubes:

- 1.5 mL microcentrifuge tube (for PCR master mix)
- 2 mL SC micro tube PCR-PT (Starstedt 72694.406) (for dual index primers).

Pipette tips:

- Master mix aliquoted with 175 µL filtered non-conductive (PerkinElmer).
- All others are 25 µL filtered non-conductive (PerkinElmer).

The PCR master mix in a 1.5 mL microcentrifuge tube is aliquoted into a 96-well half skirt PCR microplate (Axygen Scientific). Dual index primers in 2 mL SC micro tube PCR-PT (Starstedt) are arrayed into the 96-well half skirt PCR microplate (see microplate layout for setup). DNA from tagmentation reaction in 384-well microplate (4titude FrameStar) is then transferred into 96-well half skirt PCR microplate. A user supplied csv dictates the volume oligo, water and oligo array destination.

5. Pooling

Program and file:

- pooling_variable_volumes_96well_plate_to_eppendorf.MPT
- pooling_variable_volumes_96well_plate_to_eppendorf_example.csv

Microplates:

- Libraries: 96-well half skirt PCR microplate (Axygen Scientific PCR-96M2-HS-C).

Microcentrifuge tubes:

- 1.5 mL microcentrifuge tube.

Pipette tips:

- 25 µL filtered non-conductive (PerkinElmer).

Sequencing libraries in 96-well half skirt PCR microplate (Axygen Scientific) are transferred to a 1.5 mL microcentrifuge tube. A user supplied csv dictates of each volume added.

Microplate layout for dual index primers

DESIRED LAYOUT OF THE 96-WELL BARCODE OLIGO PLATE

		B-0055	B-0056	B-0057	B-0058	B-0059	B-0060	B-0061	B-0062	B-0063	B-0064	B-0065	B-0066	
	**example**	**1**	**2**	**3**	**4**	**5**	**6**	**7**	**8**	**9**	**10**	**11**	**12**	
B-0031	**A**													WGS_i5_3
B-0032	**B**													WGS_i5_139
B-0033	**C**													WGS_i5_197
B-0034	**D**													WGS_i5_321
B-0035	**E**													WGS_i5_480
B-0036	**F**													WGS_i5_566
B-0037	**G**													WGS_i5_577
B-0038	**H**													WGS_i5_651

NUMBERING LAYOUT OF THE 24-RACK

B-0031	B-0035	9	B-0055	B-0059	B-0063
B-0032	B-0036	10	B-0056	B-0060	B-0064
B-0033	B-0037	11	B-0057	B-0061	B-0065
B-0034	B-0038	12	B-0058	B-0062	B-0066

NUMBERING LAYOUT OF THE 24-RACK

1	5	9	13	17	21
2	6	10	14	18	22
3	7	11	15	19	23
4	8	12	16	20	24
