# Supplementary material for: Cost-conscious generation of multiplexed short-read DNA libraries for whole-genome sequencing
Source: PLoS One. 2023 Jan 27;18(1):e0280004. doi: 10.1371/journal.pone.0280004 (PMC9882895; doi:10.1371/journal.pone.0280004)
Supplement: S2 File — (ZIP) [file pone.0280004.s002.zip › supplemental/readme.pdf]

<u>Summary of supplemental</u>

Cost-conscious generation of multiplexed short-read DNA libraries for whole-genome sequencing

Ashley Jones, David Stanley, Scott Ferguson, Benjamin Schwessinger, Justin Borevitz, Norman Warthmann

July 2022

The supplemental contains the following:

custom_dual_index_primers.xlsx
Custom DNA oligos for dual index primers during PCR. Enables multiplexing of nine 96-well microplates, whereby up to 864 samples can be placed on a single flow cell if required.

library_costs_per_reaction.xlsx
Comparison of protocol prices, per library reaction. Our presented cost-conscious protocol compared to Illumina DNA Prep protocol.

program_files_PerkinElmer_workstations (directory)
Program files for automated workstations by PerkinElmer. Includes files for JANUS NGS Express and JANUS G3. A summary of the equipment and consumables required is also included. Microsoft excel files provided can be altered to change the volumes pipetted in certain programs.

microplate_reader_concentrations (directory)
Microsoft excel sheets for calculating the concentration of samples when using a fluorescent microplate reader. Excel sheets to analyse readings from a FLUOstar Omega (BMG Labtech) and Infinite M1000 PRO (Tecan) are provided.
